# Age-Related Differences in Lexical Access Relate to Speech Recognition in Noise

**DOI:** 10.3389/fpsyg.2016.00990

**Published:** 2016-07-04

**Authors:** Rebecca Carroll, Anna Warzybok, Birger Kollmeier, Esther Ruigendijk

**Affiliations:** ^1^Cluster of Excellence ‘Hearing4all’, University of OldenburgOldenburg, Germany; ^2^Institute of Dutch Studies, University of OldenburgOldenburg, Germany; ^3^Medizinische Physik, University of OldenburgOldenburg, Germany

**Keywords:** age, speech perception in noise, mental lexicon, lexical access, vocabulary size, verbal working memory, cognitive change

## Abstract

Vocabulary size has been suggested as a useful measure of “verbal abilities” that correlates with speech recognition scores. Knowing more words is linked to better speech recognition. How vocabulary knowledge translates to general speech recognition mechanisms, how these mechanisms relate to offline speech recognition scores, and how they may be modulated by acoustical distortion or age, is less clear. Age-related differences in linguistic measures may predict age-related differences in speech recognition in noise performance. We hypothesized that speech recognition performance can be predicted by the efficiency of lexical access, which refers to the speed with which a given word can be searched and accessed relative to the size of the mental lexicon. We tested speech recognition in a clinical German sentence-in-noise test at two signal-to-noise ratios (SNRs), in 22 younger (18–35 years) and 22 older (60–78 years) listeners with normal hearing. We also assessed receptive vocabulary, lexical access time, verbal working memory, and hearing thresholds as measures of individual differences. Age group, SNR level, vocabulary size, and lexical access time were significant predictors of individual speech recognition scores, but working memory and hearing threshold were not. Interestingly, longer accessing times were correlated with better speech recognition scores. Hierarchical regression models for each subset of age group and SNR showed very similar patterns: the combination of vocabulary size and lexical access time contributed most to speech recognition performance; only for the younger group at the better SNR (yielding about 85% correct speech recognition) did vocabulary size alone predict performance. Our data suggest that successful speech recognition in noise is mainly modulated by the efficiency of lexical access. This suggests that older adults’ poorer performance in the speech recognition task may have arisen from reduced efficiency in lexical access; with an average vocabulary size similar to that of younger adults, they were still slower in lexical access.

## Introduction

Speech perception in background noise is relatively difficult compared to speech perception in quiet, and it most likely depends on a conglomerate of multiple factors (e.g., Benichov et al., 2012; Humes et al., 2012; Füllgrabe et al., 2015). Acoustic-perceptual factors such as pure-tone thresholds and acoustic setting, e.g., masker type, spatial configuration, or the signal-to-noise ratio (SNR), are among the most obvious candidates. Speech recognition is well-documented to deteriorate with decreasing SNR (Plomp and Mimpen, 1979; Kollmeier and Wesselkamp, 1997; Bruce et al., 2013). But cognitive factors such as (verbal) working memory, sensitivity to interference, attention, processing speed, or adaptive learning have also been shown to contribute to speech perception in noise (Pichora-Fuller, 2003; Pichora-Fuller and Souza, 2003; Füllgrabe and Moore, 2014; Füllgrabe, 2015; Heinrich et al., 2015; Huettig and Janse, 2016). Age has been reliably found to alter speech perception on top of that, although the exact mechanisms are not completely understood (see overviews by CHABA, 1988; Wayne and Johnsrude, 2015). Linguistic factors including lexical access, inhibition of lexical competitors, and integration of phonemic and lexical information into context (e.g., Cutler and Clifton, 2000; Weber and Scharenborg, 2012) are important for speech processing in general. Although not all linguistic factors have been tested in acoustically challenging conditions or in older populations, speech perception in noise is likely to follow known mechanisms of speech perception and word recognition that are known for speech presented in quiet. We consider various measures that are applied to determine individual differences: hearing levels, age, working memory, vocabulary size (i.e., how many words a person knows), and lexical access time. The umbrella term ‘individual difference measures’ refers to the collection of all of these measures.

Deterioration of speech recognition in noise is characteristic of older listeners, even in individuals with normal or almost normal pure-tone thresholds (CHABA, 1988; Dubno et al., 2002; Pichora-Fuller, 2003; Zekveld et al., 2011; Schoof and Rosen, 2014; Besser et al., 2015). Possible explanations for this deterioration vary from age-related changes in supra-threshold auditory processing (Legér et al., 2012; Schoof and Rosen, 2014; Füllgrabe et al., 2015) to age-related changes in cognitive factors such as processing speed, working memory, and susceptibility to interference (e.g., Füllgrabe et al., 2015; see also Wayne and Johnsrude, 2015; Wingfield et al., 2015 for recent reviews). Füllgrabe et al. (2015), for example, observed age effects on speech-in-noise recognition in English and explained these by age-linked reductions in sensitivity to temporal fine structure and a composite measure of cognition. Self-rated hearing ability and modulation masking release did not explain age-related differences in speech recognition.

On the cognitive level, speech recognition has been suggested to rely strongly on working memory (e.g., overview by Besser et al., 2013). The general assumption is that the larger the working memory is, the better the speech recognition scores (see Rudner and Signoret, 2016). The Ease of Language Understanding (ELU) model (Rönnberg et al., 2013) posits that a degraded speech input is not automatically matched against a semantic representation in long-term memory. This mismatch in the rapid automatic multimodal phonological buffer inhibits immediate lexical access, and requires an additional, explicit processing loop. Crucially, this explicit semantic processing loop is thought to depend on working memory, because the phonetic details of the input signal and the semantic content of the context have to be held in short-term memory (or the phonological buffer) while searching for a lexical match. The ELU model suggests that successful perception of degraded speech necessitates relatively more selective attention at very early stages of stream segregation and relatively more working memory capacity to match the degraded input with long-term representations in the mental lexicon. The model also suggests that the influence of cognitive factors on speech recognition increases as the speech signal deteriorates, e.g., due to decreasing SNRs (see e.g., Rudner et al., 2012). Another way of thinking about the relation between cognition and speech perception in adverse conditions is provided by the cognitive spare capacity hypothesis (Mishra et al., 2013, 2014). Assuming that working memory is limited (Baddeley and Hitch, 1974), cognitive spare capacity is defined as the resources that are still available after those cognitive capacities required for lexical access have been recruited. Since listening in adverse conditions, such as noise and/or hearing impairment, is assumed to require more cognitive resources to match a stimulus to the semantic long-term representation (see Rönnberg et al., 2013), comparatively less cognitive spare capacity for post-lexical speech processing and integration into discourse is expected in these situations than in acoustically less challenging situations. Mishra et al. (2013, 2014) could show that cognitive spare capacity was somewhat independent of working memory, but was related to episodic long-term memory. Despite a growing body of published evidence supporting the cognitive spare capacity hypothesis, the ELU model, and the importance of working memory in general, higher working memory capacity has not been universally found to benefit speech recognition in acoustically adverse conditions. Rudner et al. (2012), for example, found that perceived listening effort ratings correlated with speech perception in different types of noise and at different SNRs, but were independent of working memory capacity for two groups of Danish and Swedish listeners with hearing loss. Working memory did not influence speech perception *per se* but seemed to influence the relative rating of perceived effort with respect to different noise types. Picou et al. (2013) presented US-American hearing-impaired listeners with a dual-task comprising a word recognition task and a visual reaction time (RT) task. Working memory capacity was related to a word recognition benefit from visual cues, but was not associated with changes in the auditory presentation (i.e., the addition of noise).

Speech recognition in acoustically adverse conditions has also been suggested to depend on linguistic factors, such as vocabulary knowledge. Benard et al. (2014) reported a positive correlation of phoneme restoration scores with vocabulary size as measured by the Dutch version of the Peabody Picture Vocabulary Test (PPVT; Bell et al., 2001), suggesting that the more words a listener knows, the better his/her speech recognition scores in a phoneme restoration task. McAuliffe et al. (2013) observed a correlation between the PPVT and recognition scores for English dysarthric speech, supporting the idea that a large lexicon may be beneficial for word recognition in adverse listening conditions. Benichov et al. (2012), in contrast, observed no relation between vocabulary knowledge and speech recognition in English sentences with predictable (vs. unpredictable) final words. They concluded that their participants’ (aged 19–89) ability to benefit from the predictability of linguistic context was “sufficiently robust that a relatively wide range in verbal ability among native English speakers had no effect on [speech] recognition performance” (Benichov et al., 2012, p. 250). Banks et al. (2015) investigated effects of inhibition, vocabulary knowledge, and working memory on perceptual adaptation to foreign-accented English speech. Vocabulary knowledge predicted better recognition of unfamiliar accents, whereas working memory only indirectly influenced speech recognition, mediated by listeners’ vocabulary scores. Research on the role of vocabulary size across the adult life span diverges even more. Despite the cumulating support for an association of vocabulary knowledge with speech recognition in adverse conditions, the reasons and associated mechanisms are far from understood, especially with respect to the standardized tests that are typically used. For example, why should knowing relatively obscure words from vocabulary tests (e.g., *usurp* or *concordance*) predict correct recognition and recall of relatively familiar words such as *clouds* or *table* that are typically used in standardized speech recognition tests? McAuliffe et al. (2013) cautiously suggested that a larger vocabulary size may require a more fine-grained or detailed lexical representation. They did, however, not test this hypothesis. To confirm the impact of vocabulary knowledge on speech recognition in noise, Kaandorp et al. (2015) compared speech-in-noise recognition scores of three groups of normal-hearing young listeners with their vocabulary knowledge and lexical access. Although vocabulary knowledge and lexical access were highly correlated, only lexical access times reliably predicted speech recognition scores: faster lexical access correlated with better speech recognition scores.

Unfortunately, there is no compelling theory that can explain all of the above observations. The ELU model (Rönnberg et al., 2013) provides a reasonable explanation to delineate the relation of working memory and disturbed lexical access due to mismatch. It does not, however, allow a direct prediction of how vocabulary size would modulate this mismatch (McAuliffe et al., 2013; cf. Benard et al., 2014). The speculation is that the more words someone knows, the more likely a lexical match is (or the less likely a mismatch that would trigger the explicit phonological or semantic processing loop). As a consequence, word recognition should be faster in people with larger lexicons. Considering the findings by Banks et al. (2015), it is possible that both vocabulary knowledge (or lexical representation) and working memory may only *indirectly* relate to speech recognition (in noise-distorted speech). Lexical access times may mediate the explanatory gap between lexical representations in the lexicon (either word form or semantic knowledge) and successful speech recognition, especially in acoustically adverse conditions (Kaandorp et al., 2015). The faster—or rather the more efficient—a person’s lexical access, the better the corresponding speech recognition score because that leaves more spare capacity for resolving acoustic-phonetic matching difficulties of subsequent speech material, or for integrating recognized speech into discourse context (e.g., Mishra et al., 2013, 2014; Rönnberg et al., 2013). There are, however, at least two problems with the simplistic prediction of a large vocabulary size leading to fast lexical processing and integration into sentence context, thus resulting in successful speech-in-noise recognition: (A) There is evidence from bilingualism and aging studies suggesting that a larger lexicon may require longer search times, resulting in slower speech processing times (e.g., Vitevitch and Luce, 1998; Salthouse, 2004; Ramscar et al., 2014; Schmidtke, 2014). (B) The role of age-related differences in vocabulary knowledge, lexical access time, and working memory is somewhat obscure. Age is not only associated with changes in speech recognition in noise as pointed out above, but also with changes in cognitive and possibly linguistic factors. Several authors have posited that vocabulary knowledge increases with increasing age (e.g., MacKay and Burke, 1990; Kavé and Halamish, 2015; Keuleers et al., 2015). Others reported peak vocabulary knowledge with subsequent decline in later adulthood (e.g., Salthouse, 2004; Kavé et al., 2010; Hartshorne and Germine, 2015). Are age-related differences in vocabulary knowledge, lexical access time, and working memory comparable, and how do they relate to word recognition? Older adults have been found to have lower working memory capacities than younger adults (e.g., Pichora-Fuller et al., 1995; Desjardins and Doherty, 2013; Kidd and Humes, 2015). However, whether working memory capacity relates directly or indirectly to generally poorer speech processing or language understanding, remains unclear (see e.g., Besser et al., 2013; Banks et al., 2015). Ramscar et al. (2014) argued that a larger lexicon requires more detailed representations to allow efficient lexical access. They further proposed that older adults underperform in word recognition tests, because they know more words than younger adults, which may require longer searches. This lexical access account for age-related differences in word recognition may be independent of or in addition to age-related declines in cognitive skills. Accordingly, people with a larger lexicon should perform worse in speech recognition tasks, unless they can compensate with better working memory (ELU model, Rönnberg et al., 2013).

Based on the findings from different languages as described above, vocabulary knowledge likely contributes to higher speech recognition scores, but it may be mediated by lexical access and possibly working memory. We contribute to the existing research by adding data from a German population, and by focusing on a theoretically motivated explanation that takes into account the intricate interplay of known factors that contribute to speech recognition. We hypothesized that only the *relative* efficiency of lexical access may be correlated with successful speech recognition. Relative efficiency comprises a combination of vocabulary size and lexical access times: quick lexical access relative to the vocabulary size should predict good speech recognition scores. Slow access relative to the vocabulary size should predict worse recognition. Provided that listeners have no time constraints, a slow-but-detailed approach may be acceptable but is not efficient. To perform well in speech recognition, slow access listeners would have to be able to keep the word(s) in their phonological loop for rehearsing instead. Such an explanation could thus coherently integrate all of the individual difference measures listed above. The focus of our investigation is on the role of the mental lexicon for success in a standardized speech-in-noise recognition test. We submit that the efficiency with which the lexicon is accessed may modulate speech recognition performance. We attempt to show this using a limited battery of tests that can be administered in a clinical routine. Whereas many studies reporting hearing status or hearing device strategies as the key predictor of speech recognition difficulties amalgamate young and older adults (e.g., Dirks et al., 2001; George et al., 2006; Mackersie et al., 2015), we attempt to tease apart influences of age-related and hearing-related differences for speech perception in noise.

Our specific research questions were:

• Do individual difference measures pertaining to cognitive-linguistic aspects change with age? If so, which factors and in what way?
• Which of these individual difference measures are relevant predictors of speech-in-noise recognition performance?
• Can age-related differences in the efficiency of lexical access explain differences in speech recognition scores?

## Materials and Methods

### Participants

Two groups of native listeners of German with normal hearing participated in the experiments. The first group consisted of 22 younger listeners (YNH, 13 women and 9 men), varying in age from 18 to 35 years, with an average age and corresponding standard deviation of 25.3 ± 4.1 years. The second group included 22 older listeners (ONH, 15 women and 7 men), who ranged in age from 60 to 78 years, with an average age of 67.7 ± 4.8 years. Inclusion criteria were based on normal hearing status (see section “Hearing Status” for a detailed description) and age group (YNH: 18–35, ONH: 60–80). Education may play an indirect role in speech recognition because people with advanced education may know more words (see also Kaandorp et al., 2015), and highly educated people may also be better with respect to working memory capacity, adaptability, and lexical access time. Education level was assessed using a questionnaire: participants were categorized according to their highest level of education (doctoral, master’s, bachelor, high school, or middle school degree).

### Speech Recognition Task

We tested speech-in-noise recognition with the Göttingen Sentence Test (GÖSA; Kollmeier and Wesselkamp, 1997), which consists of short, meaningful sentences from everyday speech with a structure similar to the Plomp-type sentences (Plomp and Mimpen, 1979) or the Hearing in Noise Test sentences (Nilsson et al., 1994). GÖSA sentences vary with respect to their syntactic complexity, from simple subject-verb-object sentences to more complex structures (see Uslar et al., 2011). They also vary with respect to the context-driven predictability of individual words. For example, *Spiele* ‘games’ in *Er gewinnt vier Spiele nacheinander*, ‘He wins four games in a row’ is highly predictable, while *Licht* ‘light’ in *Mach doch das Licht an* ‘Do turn on the light’ is not as predictable. The sentences with varying complexity and context are distributed equally across test lists. The test is optimized and evaluated for speech intelligibility in noise (Kollmeier and Wesselkamp, 1997). The test contains 10 statistically and phonemically balanced lists, each of 20 sentences.

### Individual Difference Measures

#### Hearing Status

**FIGURE 1:**
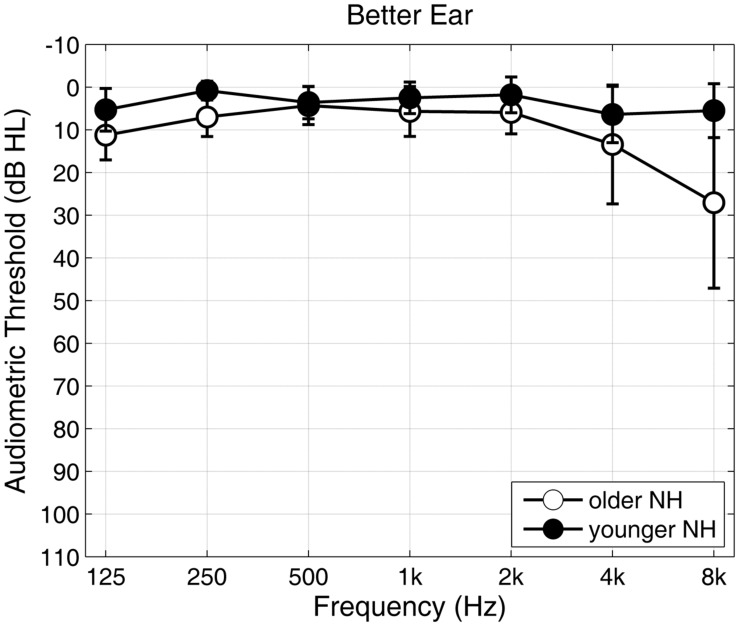
**Audiogram for younger (YNH, solids) and older (ONH, circles) listeners with normal hearing.** Mean pure-tone hearing thresholds and standard deviations are presented for octave frequencies from 125 Hz to 8 kHz of the better ear.

YNH listeners were defined as having normal hearing when their pure-tone thresholds were equal to or better than 20 dB HL across the octave frequencies from 125 Hz to 8 kHz in the better ear. This strict criterion was relaxed for the group of ONH listeners. Age-related changes in pure-tone threshold mostly affected frequencies of 4 kHz and above. The main speech-relevant frequency range between 500 Hz and 4 kHz remained, however, unaffected. ONH were therefore defined as having normal hearing when their pure-tone average across the frequencies 500, 1k, 2k, 4k Hz (PTA-4), was equal to or lower than 20 dB HL in the better ear. The average PTA-4 was 3.2 ± 3.0 dB HL for the YNH group and 8.4 ± 4.5 dB HL for the ONH group. Differences in hearing level between the better and the worse ear were 15 dB HL or less at each of the PTA frequencies, except for 3% of the data, where the interaural difference was higher in maximally one PTA frequency per person. **Figure 1** shows the mean pure tone thresholds with corresponding standard deviations for the YNH (solid dots) and ONH (circles) group at the better/measured ear. Given that most of the spectral power of the GÖSA speech material was between 100 Hz and 5 kHz, small differences in hearing level at or above 4 kHz were not expected to cause notable differences in speech recognition.

#### Verbal Working Memory

Verbal working memory was tested using the German version of the *Reading Span Test* (RST) that has been suggested for application in cognitive hearing research (Carroll et al., 2015). This test consists of 54 short sentences, half of which are semantically sensible and half of which are absurd. The participants’ task was to read a sentence presented on a screen and to indicate via button press within 1.75 s whether the sentence was absurd or not. After a block of 3, 4, 5, or 6 sentences, participants were asked to repeat either the first noun or the last word of each sentence in that block. Correctly recalled items in the correct order were scored on a sheet of paper.

#### Lexical Access Times

The *Lexical Decision Test* (LDT) presented four-letter combinations on a computer screen. Forty items were monosyllabic pseudowords (i.e., non-existent words that are structurally possible but carry no meaning in German, e.g., MAND). Forty items were monosyllabic existing words, of which half (*n* = 20) occur frequently and half occur infrequently in the language. Frequency of occurrence was established using the Leipzig Wortschatz corpus^1^ The participants’ task was to decide as quickly and as correctly as possible whether a given letter combination represented an existing German word. Responses were collected via button press. Presentation and logging was done using the E-Prime 2.0 professional software (Psychology Software Tools, Inc., Pittsburgh, PA, USA). RTs were calculated for correctly answered trials. Lexical access time was defined as the mean RT of all words with both high and low frequency of occurrence. Frequent words are more likely to be pre-activated than less frequent words (e.g., Marslen-Wilson, 1989; Cleland et al., 2006); RT are therefore bound to be much faster. On the flipside, RT to infrequent words may be more strongly influenced by vocabulary size than RT to frequently used words. For our main analyses, we therefore averaged RT to all words, log-transformed them to minimize the effects of long latencies, and z-transformed for the statistical analyses to allow for direct comparisons across listener groups and with other tests. To check for response biases or speech-accuracy tradeoffs (SATs), we also analyzed RT to frequent and infrequent words separately.

#### Vocabulary Size

Two standardized tests of receptive vocabulary size were measured, the *Wortschatztest* (WST; Schmidt and Metzler, 1992) and an updated German version of the PPVT (Buhlheller and Häcker, 2003). The use of standardized tests of vocabulary size follows other studies that found good correlations with sentence in noise recognition (e.g., McAuliffe et al., 2013; Benard et al., 2014; Kaandorp et al., 2015).

In the *WST*, participants were presented 42 lines of six words each on a sheet of paper. Five of these words per line were pseudowords and one was an existing word. The task was to identify the existing word in each row at the necessary pace. Participants were instructed to not mark anything unless they were sure they could recognize the existing word. We assume the WST to test recognition of the (orthographic) word form. Semantic knowledge is not required (albeit beneficial) for high scores.

In the *PPVT*, participants saw four pictures on a paper test block and heard a target word from a loudspeaker. The task was to indicate the picture that best represented the target word. Responses were not timed. The test consists of 89 trials with increasing picture-matching difficulty. To perform well on this test, individuals not only needed to be familiar with the (acoustic) word form but also have a detailed semantic representation of the target word to correctly distinguish the correct picture from its three semantically similar and/or related competitors. We therefore assume that the PPVT focuses more strongly on semantic representations (and/or world knowledge) compared to the WST. Note that guessing and competitor elimination strategies cannot be completely excluded, especially in the PPVT.

To reduce test-specific effects and to focus exclusively on vocabulary size, we combined the z-transformed scores of the WST and PPVT into a new composite variable VOCABULARY, which we used for further analyses (see Salthouse, 2010, p. 105; Schoof and Rosen, 2014 for similar procedures). In addition, relatively higher error rates for detecting infrequent existing words (LDT_LFerror_) on the lexical decision task may also arguably reflect vocabulary size: the fewer words a person knows, the more likely he/she is to reject an infrequent word as a pseudoword in the LDT. We therefore also considered LDT_LFerror_ as a potential factor reflecting vocabulary size.

### Procedure

All GÖSA stimuli were presented using the Oldenburg Measurement Application (HörTech gGmbH, Oldenburg, Germany^2^) and free-field equalized Sennheiser HDA200 headphones. They were amplified by either an Earbox 3.0 High Power (Auritec, Hamburg, Germany) or an RME Fireface UCX (RME, Haimhausen, Germany). Measurements took place in a sound-attenuated booth that fulfilled the requirements of ANSI/ASA S3.1 and S3.6 standards (ANSI, 1999). The headphones were free-field equalized (ISO, 2004) using a finite impulse response filter with 801 coefficients. The measurement setup for speech intelligibility measurements was calibrated to 65 dB SPL using Brüel and Kjær artificial ear type 4153, the microphone type 4134, preamplifier type 2669, and amplifier type 2610 (Brüel and Kjær, Nærum, Denmark). PPVT words were presented using pre-recorded soundfiles over a Genelec 8020 loudspeaker. Signals in the speech recognition measurements were presented diotically to the better ear at fixed SNRs of -4 and -6 dB, with the test-specific noise signal fixed at 65 dB SPL. SNRs were chosen to correspond to SRTs yielding 50 and 80% intelligibility for young adults with normal hearing (see Kollmeier and Wesselkamp, 1997). The noise signal was turned on 500 ms before and turned off 500 ms after presentation of each sentence. In addition, 50 ms rising and falling ramps were applied to the masker using a Hann window, to prevent abrupt signal onset and offset. The listeners’ task was to repeat the words they had understood, and the test instructor marked the correct responses on a display; each word in a sentence was scored separately. Each participant listened to six test lists: one test list presented speech in noise at -4 dB SNR and one list at -6 dB SNR; the other four test lists presented sentences in other acoustic settings that are not under investigation here. The order of test list and acoustic condition was randomized. This study was approved by and carried out in accordance with recommendations from the local ethics committee at the University of Oldenburg.

### Statistical Analyses

To address our hypotheses, we conducted several different analyses. (1) To determine age-related group differences in our individual difference measures, we performed a multivariate analysis of variance (MANOVA). Since, in theory, age effects on all predictors may be independent from speech recognition, we excluded that latter factor here. As mentioned above, we combined WST and PPVT scores to a new composite variable VOCABULARY, to reduce test-specific effects and to avoid collinearity. (2) To appropriately model our dataset statistically, and to assess the relevant factors contributing to speech recognition, we employed an overall linear mixed effects regression (lmer) model using the lme4 package (Bates et al., 2014) in R 3.1.0. This model is described below. (3) To determine whether speech recognition scores were differentially associated with cognitive-linguistic measures depending on age group and/or SNR level, we applied hierarchical forward regression models for each listener group and for each SNR level. These planned *post hoc* analyses were based on the lmer outcome (see also Heinrich et al., 2015 for a similar approach). Five YNH participants did not complete the SNR-4 test list and the RST because the test protocol was expanded to include a measure at higher intelligibility (∼80% correct) and a measure of working memory after some first measurements. Numbers of participants are provided for each analysis. For the lmer model, we applied an exploratory approach in determining the need for random and fixed effects. Because the individual measures used very different scales, they were z-transformed for direct comparisons. The necessity to include random intercepts for LISTENER and LIST was assessed to account for possible variability (for each listener and each GÖSA list) in the effects of certain predictors. The following fixed factors (predictors) were considered in order to determine the best-fitting model: AGEGROUP (YNH vs. ONH), SNR level (-4 vs. -6 dB SNR), CONDITIONORDER, AGE, PTA-4, RST, RT in the lexical decision test (LDT_RT_), error rate for infrequent words in the lexical decision test (LDT_LF error_), VOCABULARY, EDUCATION. Testing the possible predictors GROUP, SNR level, RST, LDT_RT_, VOCABULARY, and education followed directly from our hypotheses. CONDITIONORDER (i.e., the order in which SNR-6, SNR-4, and the four unrelated acoustic conditions were presented) was considered as a factor in order to account for possible training or adaptation effects. We also considered different interactions. The model improvement for adding each predictor was determined by comparing the Akaike Information Criterion (AIC; Akaike, 1974) of the simpler and the more complex model. A significant reduction (of at least 2) in the AIC indicates that the higher model complexity (added predictor) compared to the simpler model is warranted (see Janse, 2009; Baayen and Milin, 2010). By introducing a penalty term for the number of parameters in the model, AIC resolves the danger of improving model likelihood by adding too many predictors.

## Results

After first data inspection, we determined which individual difference measures change with age (section “How Do Individual Difference Factors Change with Age?”), then determined which of these measures actually explain variance of our speech in noise task (section “Which Factors Relate to Speech Recognition in Noise?”), and finally tested whether age-related differences in the individual difference measures relate to the age-related differences for the speech recognition task (section “Can Age-Related Differences in Lexical Access Efficiency Explain Speech Recognition Scores?”). **Table 1** summarizes the results and descriptive statistics of all variables for both listener groups.

**Table 1:** Summary of descriptive statistics for the individual differences measures for younger (YNH, *N* = 22) and older (ONH, *N* = 22) listeners with normal hearing thresholds.

Function	Test	Levene’s *F*(1,42) ‡*F*(1,36)	YNH			ONH		
				
			Mean ± SD	Range	K-S	Mean ± SD	Range	K-S
Hearing level	PTA-4	3.05	3.24 ± 3.03	-1.25 to 11.25	0.19*	8.36 ± 4.54	2.5–17.5	0.13
Speech recognition	‡SNR-4	‡7.12^∗^	*85.4 ± 10.60	54.6–97.2	0.17	58.9 ± 18.3	19.9–82.1	0.24*
	SNR-6	0.08	50.9 ± 16.10	18.3 – 74.1	0.24*	30.3 ± 17.9	5.3 – 70.8	0.12
Working memory	‡RST_(max.54)_	‡0.59	*24.88 ± 7.95	9–40	0.17	22.18 ± 5.96	8–32	0.08
Vocabulary size	WST_(max.42)_	0.06	32.3 ± 4.09	24–38	0.11	34.0 ± 4.81	19–39	0.11
	PPVT_(max.89)_	4.10^∗^	74.32 ± 8.35	44–89	0.29*	78.77 ± 7.36	49–89	0.34*
	LDT_LF errors_	10.22^∗^	5.36 ± 3.50	0–13	0.22*	1.82 ± 1.80	0–7	0.22*
Lexical access time	LDT_logRT_	1.40	2.70 ± 0.58	2.75–2.93	0.09	3.01 ± 0.06	2.90–3.16	0.18


**N* = 44; ^‡^*N* = 37; Levene’s test for homogeneity of variance; K-S = Kolmogorov–Smirnov test for normal distribution; ^∗^*p* ≤ 0.05.*

Speech recognition scores were about 25–30% lower for ONH than for YNH listeners. Despite their clinical status of normal hearing, and despite the fact that most of the age-related hearing loss was found in higher frequencies, PTA-4 was about 5 dB higher for ONH than for YNH. Vocabulary size was larger for ONH as indicated by higher PPVT scores and lower LDT error rates for less frequently used words. Lexical access time was slower, and working memory was about two points lower, than for YNH.

**Table 2** provides an overview of the inter-correlations between the factors in **Table 1.** Age, as a continuous variable, significantly correlated with PTA, lexical access time, and LDT_LF errors_. As the two measures of vocabulary size, WST and PPVT were highly correlated, their combination into a common variable VOCABULARY (see Materials and Methods) was deemed justified.

**Table 2:** Inter-correlations between predictor measures.

	Age	zPTA	Education	zRST^#^	LDT_RT_	LDT_LF errors_	zWST
zPTA	0.57**						
Education	-0.03 *n.s.*	0.09 *n.s.*					
zRST^#^	-0.22 *n.s.*	-0.17 *n.s.*	0.38^∗^				
LDT_RT_	0.86**	0.32*	0.11 *n.s.*	-0.36^∗^			
LDT_LF errors_	-0.55**	-0.12 *n.s.*	-0.37^∗^	-0.23 *n.s.*	-0.42^∗^		
zWST	0.01 *n.s.*	-0.01 *n.s.*	-0.57^∗∗^	0.54^∗∗^	-0.18 *n.s.*	-0.57^∗∗^	
zPPVT	0.29 *n.s.*	0.11 *n.s.*	-0.47^∗∗^	0.38^∗^	-0.18 *n.s.*	-0.51^∗∗^	0.79^∗∗^


*^∗^*p* < 0.05, ^∗∗^*p* ≤ 0.01, Pearson’s *r* and two-tailed *p-*values are reported, ^#^*N* = 39.*

### How Do Individual Difference Factors Change with Age?

Whether the individual measures listed in **Table 1** significantly differed between ONH and YNH was tested by means of a MANOVA. **Table 3** summarizes the statistical results and indicates how well the individual factors can explain the observed variances that are described in **Table 1.**

**Table 3:** Results of MANOVA group effects for individual differences besides speech recognition.

Test	*F*	*df1, df2*	*p*	*Corrected R^2^*
Age	805.36	1, 36	<0.001***	0.956
zPTA-4	16.06	1, 36	<0.001***	0.289
zRST‡	1.08	1, 36	0.305 *n.s.*	0.002
Vocabulary	0.33	1, 36	0.569 *n.s.*	-0.018
LDT_LF errors_	23.76	1, 36	0.001***	0.261
LDT_logRT_	14.08	1, 36	<0.001***	0.712
Education	0.26	1, 36	0.610 *n.s.*	-0.020


**N* = 44; ^‡^*N* = 39.*

The hearing levels of ONH were significantly higher [on average 5 dB; *F*(1,36) = 16.06; *p* < 0.001; see **Table 1**] than those of YNH, despite the fact that both groups had hearing levels in the normal range (according to World Health Organization [WHO], 2016 criteria). The difference was mainly triggered by higher thresholds at 4 kHz for older adults. Verbal working memory, as measured by z-transformed RST, did not differ significantly between the groups. We therefore assume that the working memory capacity of YNH and ONH participants varied to roughly the same degree. Both groups had similar levels of education: we could not establish a significant group effect based on participants’ highest degree of education.

An AGEGROUP effect for lexical access time (LDT_RT_) indicates that older adults were significantly slower in their lexical access than younger adults [*F*(1,36) = 14.08; *p* < 0.001]. In addition, older adults made fewer mistakes on infrequent words compared to younger adults, as evidenced by the effect for LDT_LF errors_ [*F*(1,36) = 23.76; *p* < 0.001]. This suggests that older adults knew more infrequent words than younger adults. To exclude the possibility that age-related differences in LDT_RT_ and LDT_LF error_ were merely an effect of SAT or response bias instead of an age-linked difference in vocabulary size, we correlated error rates and RT to frequent and infrequent words separately and compared them to our other vocabulary measures (see **Table 4**). A negative correlation between LDT error rates and either PPVT or WST indicates a measure of vocabulary size: the more words are known, the fewer errors should be made during lexical decision. This is independent of an SAT or response bias. As **Table 4** illustrates, Pearson’s r was highly negative for both groups (YNH: *r* = -0.73/-0.71; *p* < 0.001; ONH: *r* = -0.52; *p* < 0.05/*r* = -0.71; *p* < 0.001). The smaller correlation of LDT_LFerror_ and PPVT in ONH (*r* = -0.52) was most likely due to a generally high performance on the PPVT (*M* = 78.77 ± 7.36; about 88%). The correlation was, however, still substantially negative, indicating a similar effect in both age groups. Kaandorp et al. (2015) described an SAT or response bias for their young listeners with normal hearing: relatively fast answers elevated the probability of incorrect decisions. In our dataset, an SAT would be reflected by a strong negative correlation of RT and errors, especially for low frequency words, as these were more error prone. The correlation for ONH in **Table 4** suggests the opposite: a substantial positive correlation for infrequent words (*r* = 0.53, *p* < 0.05) indicates that infrequent words were either quickly correctly recognized, or not correctly identified even after a long search time. There may, however, have been an SAT tendency for frequent words in the ONH group. The negative correlation of RTs to frequent words and vocabulary size (*r* = -0.33; *p* > 0.05) did not reach significance to begin with, and we argue that exclusion of incorrect trials and our use of averaged RT for all words should further reduce any impact of a potential response bias.

**Table 4:** Correlation of vocabulary size measures and lexical access time per group (Pearson’s *r*).

	Young (YNH)				Old (ONH)			
		
	PPVT	WST	LDT_LFerror_	LDT_HFerror_	PPVT	WST	LDT_LFerror_	LDT_HFerror_
LDT_LF errors_	-0.73^∗∗^	-0.71^∗∗^			-0.52^∗^	-0.70^∗∗^		
LDT_LF-RT_	-0.40	-0.29	-0.05		-0.74^∗∗^	-0.82^∗∗^	0.53^∗^	
LDT_HF-RT_	0.45^∗^	0.33	-0.31	0.33	-0.46^∗^	-0.57^∗^	0.27	-0.33


*HF, high frequency; LF, low frequency; RT, reaction time; ^∗^*p* ≤ 0.05; ^∗∗^*p* ≤ 0.001.*

**FIGURE 2:**
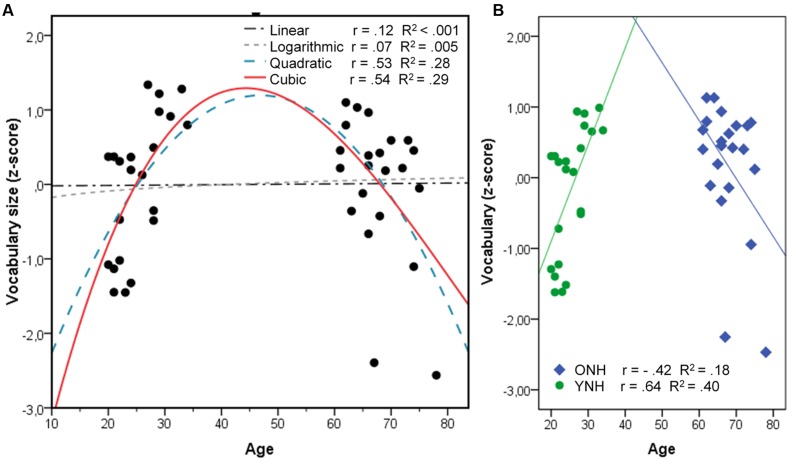
**Correlations of vocabulary size and age in assuming a linear, logarithmic, quadratic, or cubic model fit over all data **(A)**, and separate linear models for each group **(B)****.

The composite variable VOCABULARY did not show any AGEGROUP effect [*F*(1,36) = 0.33; *p* = 0.57], suggesting that both groups knew about the same number of words tested in the WST and the PPVT. **Figure 2** illustrates the distribution of VOCABULARY size over age, which appears to be non-linear. Whereas VOCABULARY size increased with age in the younger group, it seemed to decrease with increasing age in the ONH group. This observation was supported by a statistically significant correlation in a cubic distribution (*r* = 0.53; *R^2^* = 0.29; *p* = 0.003), which fit the data better than our initial linear fit (*r* = 0.12; *R^2^* < 0.001; *p* < 1). Because the model is missing data points between 35 and 60 years, using one model to fit all data points may not be appropriate. We circumvented this uncertainty by applying separate regression analyses per age group in addition to the overall analysis to determine a possible association of vocabulary size with speech recognition.

### Which Factors Relate to Speech Recognition in Noise?

Given the complex interplay of cognitive factors as indicated by previous studies, it seemed worthwhile to include several factors that have previously been identified to relate to speech recognition processes. To this end, a linear mixed effects regression model including all data points with the z-transformed dependent variable speech recognition score was built. Based on previous research, we expected main effects of AGEGROUP, SNR level, RST, vocabulary size, and lexical access times. We also tested for random effects and possible interactions.

We established LISTENER as a random factor (intercept variance = 0.133, *SE* = 0.36), allowing for individual intercepts per listener to account for individual differences. **Table 5** summarizes the best-fitting model, which includes data from 39 listeners (22 ONH, 17 YNH). The restricted maximum likelihood criterion at convergence was 133.2.

**Table 5:** Best-fitting linear mixed-effects regression model.

	*B*	*SE*	*t*	*p*
Intercept	-0.927	0.364	-2.55	0.0108 *
AGEGROUP (YNH vs. ONH)	2.160	0.348	6.22	<0.0001 ***
SNR (-6 vs. -4)	-1.158	0.091	-12.66	<0.0001 ***
CONDITIONORDER	0.100	0.030	3.33	0.0009 ***
LDT_RT_	0.669	0.191	3.51	0.0005 ***
VOCABULARY	0.297	0.110	2.71	0.0068 **
RST	0.007	0.012	0.64	0.5238 *n.s.*


*Dependent variable: GÖSA speech recognition (z-transformed); random effect: LISTENER (intercept; variance = 0.09 ± 0.30); Number of observations = 77; 39 listeners; restricted maximum likelihood criterion at convergence: 133.2; RST, reading span; SNR, signal-to-noise ratio; LDT_RT_, log-transformed reaction times of words in the Lexical Decision Test; significance levels ^∗∗∗^*p* ≤ 0.001, ^∗∗^*p* ≤ 0.01, ^∗^*p* ≤ 0.05.*

As expected, AGEGROUP was a significant predictor: the younger group (YNH) performed better in the speech-in-noise recognition task than the older group (ONH; *B* = 2.16; *t* = 6.22). Similarly, the SNR level was a strong contributor to speech-in-noise recognition: the lower SNR (-6 dB) predicted lower speech recognition scores than the higher SNR (-4 dB; *B* = -1.16; *t* = -12.66). CONDITIONORDER was a strong predictor, indicating that listeners’ speech recognition scores were significantly increased by the number of test lists they had heard prior to the one under investigation (*B* = 0.1; *t* = 3.33). Lexical access time—as measured by the LDT_RT_—also emerged as a significant predictor for our overall speech recognition scores: the positive estimate of coefficients (*B* = 0.67; *t* = 3.51) suggests that the longer our participants needed to decide whether a given letter combination was an existing word, the better their speech recognition scores. VOCABULARY size was also a significant predictor: the larger the participant’s vocabulary size, the better the corresponding speech-in-noise recognition scores (*B* = 0.297; *t* = 2.71). Working memory capacity (RST), although a significant predictor by itself, fell below significance level when either LDT_RT_ or VOCABULARY were also considered (*B* = 0.007; *t* = 0.64). We could not establish any interactions or random slopes that would have improved the regression model or that would have significantly improved the predictions for speech recognition. List number (i.e., which of the 10 GÖSA test lists) could not be established as a random factor, suggesting that recognition scores can be assumed to be equal across GÖSA test lists. Our final best-fitting model also excluded the non-significant factors education, LDT_LFerror_, and hearing level (PTA-4).

### Can Age-Related Differences in Lexical Access Efficiency Explain Speech Recognition Scores?

**FIGURE 3:**
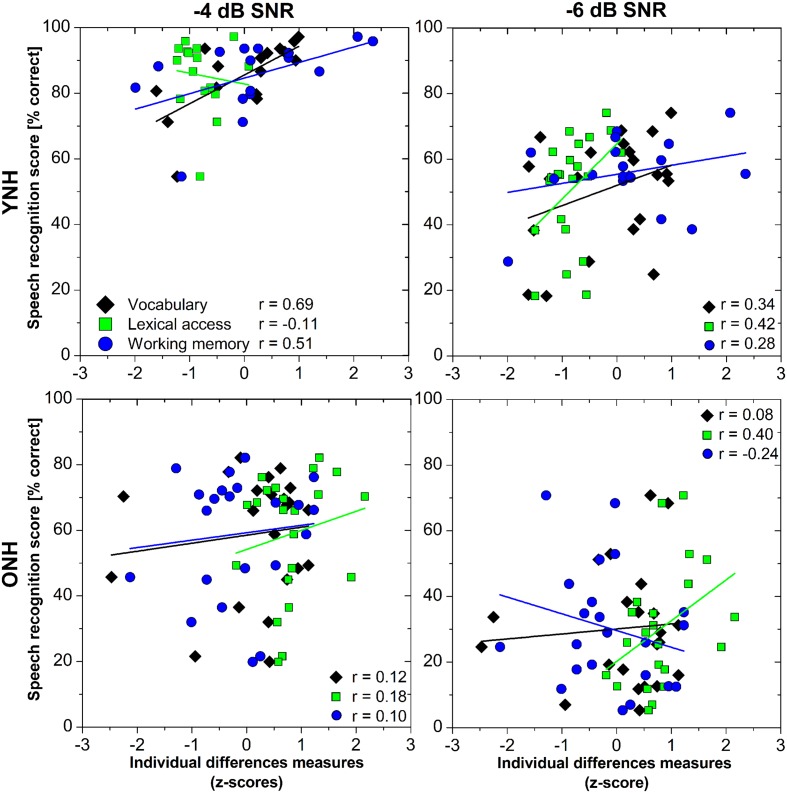
**Pearson’s correlations of speech recognition scores and individual differences measures (vocabulary, lexical access, working memory) for younger (YNH, upper panels) and older (ONH, lower panels) listeners with normal hearing, and for SNRs of -4 (left column) and -6 (right column) dB**.

We expected that the more words people knew, and the faster their lexical access, the better their GÖSA speech-in-noise recognition scores would be. The lmer model showed a large variability in speech recognition scores between younger and older adults and between SNR levels, but we did not detect any interactions between cognitive-linguistic tests and AGEGROUP (see previous section). Furthermore, our MANOVA (see section “How Do Individual Difference Factors Change with Age?”) suggested AGEGROUP effects in working memory and lexical access. Based on the non-linear distribution of vocabulary size over age (**Figure 2**), separate linear models were considered sensible. It is likely that different mechanisms or factors depending on AGEGROUP and/or SNR level are involved in speech-in-noise recognition. **Figure 3** shows correlations of vocabulary size, lexical access time, working memory, and speech recognition scores for each AGEGROUP and SNR.

By means of four hierarchical regression models carried out for each group and SNR level, we therefore assessed how vocabulary size, lexical access time, working memory, and age or hearing level modulated speech recognition in the two noise conditions. Each hierarchical regression analysis consisted of four forward models that always followed the same order: Model 1 (M1) included VOCABULARY only, model 2 (M2) added LDT_RT_ as a measure of lexical access time, model 3 (M3) comprised VOCABULARY, LDT_RT_, and RST. Model 4 (M4) finally added better ear hearing level (PTA-4) and age as possible additional factors. The overall lmer model had identified age in terms of AGEGROUP, but our findings (see **Table 4**) indicated that smaller age-related differences within the subgroups were possible. The order of inclusion was based on our expectation that efficiency of lexical access does not merely imply quick access, but rather refers to quick access that is relative to the vocabulary size (see Ramscar et al., 2014). The results of the four analyses per subset are reported in **Table 6.** Both the dependent variable speech recognition and the fixed factors used z-transformed scores. Missing values in the YNH group (SNR-4, RST) were replaced by averaged values.

**Table 6:** Results of four hierarchical linear regression models per subset of listener group and SNR level.

Subset		Factors included	*R*	Adj. *R^2^*	*SE*	*R^2^* change	*F* change	*df1, df2*	Significance *F* change
**YNH**
SNR-4	M1	VOCABULARY	0.55	0.27	7.95	0.30	8.62	1, 20	0.008^∗∗^
	M2	M1 + LDT	0.56	0.24	8.10	0.01	0.30	1, 19	0.59
	M3	M2 + WM	0.63	0.29	7.83	0.08	2.33	1, 18	0.15
	M4	M3 + PTA+Age	0.68	0.30	7.80	0.07	1.08	1, 16	0.36
SNR-6	M1	VOCABULARY	0.34	0.07	15.90	0.12	2.65	1, 20	0.12
	M2	M1 + LDT	0.53	0.21	14.71	0.17	4.37	1, 19	0.05^∗^
	M3	M2 + WM	0.54	0.18	14.96	0.01	0.36	1, 18	0.56
	M4	M3 + PTA+Age	0.61	0.18	14.98	0.08	0.99	1, 16	0.40
**ONH**
SNR-4	M1	VOCABULARY	0.13	-0.03	19.00	0.02	0.32	1, 20	0.58
	M2	M1 + LDT	0.42	0.09	17.80	0.16	3.78	1, 19	0.07
	M3	M2 + WM	0.48	0.10	17.75	0.05	1.10	1, 18	0.31
	M4	M3 + PTA+Age	0.58	0.12	17.37	0.12	1.40	1, 16	0.28
SNR-6	M1	VOCABULARY	0.08	-0.04	18.71	0.01	0.13	1, 20	0.72
	M2	M1 + LDT	0.68	0.40	14.18	0.45	15.81	1, 19	0.001^∗∗∗^
	M3	M2 + WM	0.68	0.37	14.51	0.004	0.13	1, 18	0.72
	M4	M3 + PTA+Age	0.74	0.41	14.10	0.09	1.55	1, 16	0.24


*LDT, log-transformed reaction times of words in the Lexical Decision Test; WM, working memory (z-transformed reading span scores); PTA, z-transformed averaged hearing level (0.5–4 kHz); *N* = 22 per group; missing values for YNH were replaced by mean values. Significant F changes are shaded, the trend is hatched; significance levels ^∗∗∗^*p* ≤ 0.001, ^∗∗^*p* ≤ 0.01, ^∗^*p* ≥ 0.05.*

For YNH listening to GÖSA sentences at -4 dB SNR, vocabulary size was the only factor that contributed significantly to the hierarchical linear regression model [M1; *F*(1,20) = 8.62; *p* > 0.001]. Lexical access time (M2), working memory (M3), and age or hearing level (M4) did not significantly improve the models. When listening to GÖSA sentences at -6 dB SNR, the combination of vocabulary size and lexical access time provided a significant model improvement [M2; *F*(1,19) = 4.37; *p* = 0.05]. For ONH, speech recognition at -4 dB SNR did not seem to relate to any of our factors. We did, however, observe a trend for M2: *F*(1,19) = 3.78; *p* = 0.07. At -6 dB SNR, ONH speech recognition scores were also related to the combination of vocabulary size and lexical access time [M2; *F*(1,19) = 15.81; *p* = 0.001], as seen for YNH. Inclusion of neither working memory (M3) nor PTA and age (M4) improved the models.

## Discussion

Our aim was to determine whether age-related differences in the efficiency of lexical access relate to performance differences in a standardized test of German sentence recognition in noise. To this end, we addressed three questions:

(1) Which individual difference measures pertaining to the mental lexicon change with age?
(2) Which of these factors are relevant predictors of speech-in-noise recognition performance?
(3) Can age-related differences in lexical access efficiency explain differences in speech recognition scores?

### Which Individual Difference Measures Pertaining to the Mental Lexicon Change with Age?

Our cognitive-linguistic tests revealed no significant effects of age on vocabulary size, working memory, or education. This finding for working memory stands in opposition to previous studies (e.g., Pichora-Fuller et al., 1995; Desjardins and Doherty, 2013; Schoof and Rosen, 2014; Kidd and Humes, 2015). The reasons for this contradictory finding are not clear. It is possible that the similar education levels of older and younger adults diluted any effect of working memory; the two measures were significantly correlated in our data set. Our hypothesis with respect to working memory did, however, relate mainly to its interplay with speech recognition and/or other measures, not with age *per se*. Although not a predictor itself, working memory could indirectly affect speech perception, and this could change with age (see, e.g., Banks et al., 2015). We also found an age-group effect of average pure-tone hearing level (PTA-4), despite the fact that all listeners had PTA-4s of 20 dB or better within the normal hearing range (according to World Health Organization [WHO], 2016 criteria). The observed significant 5 dB group difference in PTA-4 was therefore not expected to have a strong influence on GÖSA speech recognition scores.

The similarities in vocabulary size that we observed between younger and older listeners also contradict previous studies (e.g., MacKay and Burke, 1990; Salthouse, 2004; Kavé and Yafé, 2014; Kavé and Halamish, 2015; Keuleers et al., 2015). Performance on our vocabulary tests, especially the PPVT, was generally high. The similarities between the groups may therefore have arisen from our use of the standardized vocabulary tests, in which most listeners performed well (see Ramscar et al., 2014 for a similar reasoning). They probably also arose in part from an inappropriate linear model. Our composite VOCABULARY measure showed a clear non-linear distribution, in which vocabulary size increased linearly with increasing age for the younger group but decreased in the older group. This observation supports previous observations by Salthouse (2004), Kavé et al. (2010), and Hartshorne and Germine (2015) that vocabulary size may decrease after some peak.

Notably, we found strong age-group-related effects for lexical decision, both for errors on infrequent words and for lexical access time. Younger adults were faster than older adults, which is compatible with observations by Kavé and Yafé (2014). Age-related effects in a time-sensitive measure such as our visually presented lexical decision test could in principle result from general processing or motoric speed (see, e.g., Janse, 2009; Besser et al., 2012; Füllgrabe et al., 2015). The likelihood of simple speed as an exclusive explanation is comparatively small, however, because the relative RT difference between frequent and infrequent words was comparable in both groups. Older listeners also made fewer mistakes on infrequent words, which suggests better vocabulary knowledge. Our data are congruent with predictions made by the Transmission Deficit hypothesis (TDH) proposed by Burke et al. (1991). Although the TDH was proposed for age-linked word retrieval difficulties in speech production, it is based on MacKay’s (1987) Node Structure theory, a connectionist approach with applications in both speech production and perception. Connections between nodes in a network are reinforced through frequent, persistent, and recent exposure. This could explain why older adults in our study made fewer errors in recognizing infrequent words than younger adults (see also Kavé and Halamish, 2015). The TDH also postulates that connections between nodes may weaken with age, resulting in age-related word retrieval difficulties. Support for this assumption comes from production studies (e.g., Burke et al., 1991; Kemper and Sumner, 2001; Kavé et al., 2010). ONH’s slowed lexical access time supports the TDH assumption of an age-linked weakening of connections between word form and the corresponding meaning of a word in speech recognition. An efficiency reduction in lexical access can thus be explained by weakening of connections in aging, whereas better performance in recognizing infrequent words is explained by reinforced connections as a result of experience.

### Which Factors Are Relevant Predictors of Speech-in-Noise Recognition Performance?

We had expected a combination of age group, SNR level, pure-tone hearing thresholds, working memory, vocabulary size, and lexical access time to predict speech recognition scores. Our best-fitting lmer model showed that speech recognition scores for the German everyday-sentence-test were predicted by age group, SNR level, condition order, lexical access time, and vocabulary size. We discuss the implications of each individual predictor below.

Younger adults generally scored about 25–30% better on the speech recognition test than older adults at both fixed SNRs (4 and 6 dB). This finding follows a long list of similar observations (e.g., Dubno et al., 1984; CHABA, 1988; Pichora-Fuller et al., 1995; Pichora-Fuller and Souza, 2003; Füllgrabe et al., 2015; but see Schoof and Rosen, 2014 for relativization). Wayne and Johnsrude (2015) suggested that a generic age effect *per se* is unlikely to cause deterioration in speech perception performance. It is more likely that age mediates other perceptual (e.g., supra-threshold processing, e.g., Füllgrabe et al., 2015) and/or cognitive measures (e.g., Schoof and Rosen, 2014; Wingfield et al., 2015). Pure-tone hearing threshold was not a significant predictor in our model, but was significantly correlated with age, indicating decreasing hearing acuity with increasing age. It is possible that speech recognition processes employed by older adults may be affected by neural changes that pertain to temporal aspects, such as processing speed or temporal fine structure in auditory perception (e.g., Füllgrabe et al., 2015). The coding of information in the auditory nerve may be not as good as in young NH listeners, due to loss of synapses or degeneration of neurons with increasing age. Poor neural representation may arguably lead to poor speech recognition in noise (e.g., Anderson et al., 2011). However, these changes cannot be quantified with the pure tone threshold since only a few functioning neurons are required to detect a single tone in quiet (e.g., Stone and Moore, 2014; Bharadwaj et al., 2015; Füllgrabe et al., 2015).

Not surprisingly, GÖSA speech recognition scores increased with the higher SNR level, a fact that can be explained by the masking properties of the noise: the higher the SNR, the smaller the effect of masking. This finding is predicted by a number of speech recognition models, such as the Speech Intelligibility Index (ANSI, 1997) or the Speech Transmission Index (Steeneken and Houtgast, 1980), and supports a number of previous findings (e.g., Plomp and Mimpen, 1979; Kollmeier and Wesselkamp, 1997). A result that was unexpected but not surprising was that the order of list presentation seemed to play a role in speech-in-noise recognition. This suggests an effect of training, or rather perceptual learning, despite the fact that listeners never heard the same acoustic setting, or the same sentence more than once. Neger et al. (2014) dissociated perceptual and statistical learning of understanding noise-vocoded speech in groups of older and younger adults. Both groups showed perceptual learning.

More interestingly, our best-fitting model suggested that lexical access time (LDT_RT_) was a very relevant predictor of speech recognition (see Kaandorp et al., 2015 for a similar observation in Dutch). The longer participants needed to determine whether a letter combination was an existing word, the better their speech recognition scores were (especially at 6 dB SNR). This observation may seem somewhat counterintuitive. Both the ELU model (Rönnberg et al., 2013) and the cognitive spare capacity hypothesis (Mishra et al., 2013, 2014) predict the opposite: quick lexical access could be construed as more automatic lexical access with fewer mismatches that would require the engagement of the explicit processing loop.

Vocabulary size also predicted speech in noise recognition: the more words a listener knew, the better his or her speech-in-noise recognition scores were. Our data thus follow a number of studies in other languages that found a comparable relation between word knowledge or vocabulary size and speech recognition (e.g., Pichora-Fuller et al., 1995; McAuliffe et al., 2013; Benard et al., 2014; Banks et al., 2015; Kaandorp et al., 2015).

We propose that the correlation of slower lexical access time and better speech recognition scores need to be interpreted together with vocabulary size. Both factors contributed to speech perception independently; but both our overall lmer model and our hierarchical models per subset suggest that the combination of the two factors relate to the speech recognition data. Following the reasoning proposed by Ramscar et al. (2014), we argue that a larger lexicon may require longer search and hence access times because more competitors need to be evaluated. This may have been the case in YNH, where a slight positive relation between lexical access time for *frequent* words and vocabulary size suggests that larger lexicons tended toward longer searches (see **Table 4**). But lexical access time to *infrequent* words decreased with larger vocabulary size. In ONH, on the other hand, lexical access time decreased with increasing vocabulary size for both frequent and infrequent words. One explanation for this unexpected observation of group and frequency-related lexical access times is that a larger mental lexicon also necessitates a more detailed representation than a smaller lexicon to facilitate distinction and correct identification (see argumentation above, McAuliffe et al., 2013; Ramscar et al., 2014; Kaandorp et al., 2015). Following word recognition accounts that favor exemplar-based approaches (see Weber and Scharenborg, 2012 for an overview of different models), a larger lexicon is likely to entail more instances or variants per word. According to the TDH (Burke et al., 1991; see above), frequent or extended exposure (e.g., with age) to a word results in strengthening of the connections from word form to meaning. This would explain why our RT to frequent words were always faster than RT to infrequent words. A larger lexicon would therefore require longer search times for some words or some populations, but at the same time result in a higher chance of matching the input with one of the exemplars (see also Schmidtke, 2014; Kavé and Halamish, 2015 for a similar line of argumentation in bilinguals).

Working memory by itself was a significant predictor for German speech recognition but became insignificant once vocabulary size or lexical access were accounted for. Still, including working memory improved the fit of our lmer model. Our observation that working memory did not contribute strongly to speech recognition in noise does not quite follow the assumptions of the ELU model (Rönnberg et al., 2013) or the cognitive spare capacity hypothesis (Mishra et al., 2013, 2014). Our findings are consistent, however, with other studies that have also failed to identify working memory as a strong predictor, especially when other predictors were tested as well (e.g., Banks et al., 2015; Füllgrabe et al., 2015). An alternative option is more likely: the inter-correlations of working memory with lexical access times and vocabulary size, together with the fact that the latter were substantial predictors for speech recognition scores in our listeners, may arguably also suggest an indirect role of working memory (see Banks et al., 2015 for more compelling evidence for such a claim).

### Can Age-Related Differences in Lexical Access Efficiency Explain Differences in Speech Recognition Scores?

Given the age-related differences in speech recognition scores and in lexical access times, and the distribution of vocabulary size across the adult lifespan, we hypothesized that the relation between the cognitive-linguistic factors and speech recognition may be different for younger and older listeners. To test this, we calculated hierarchical regression models for each group. As noted above, the most likely speech recognition strategy should involve efficiency of lexical access, i.e., quick lexical access relative to vocabulary size. Speech recognition tests do, however, also allow a second ‘offline’ strategy: listeners can conceivably simply listen to the sentence and only start processing and ‘matching’ the acoustic signal with a lexical entry in their lexicon after the sentence has been completed. This strategy is likely to engage relatively more working memory because successful recall is only possible if the sentence can be kept in the phonological loop for rehearsal and matching (cf. Rönnberg et al., 2013).

For YNH, different SNRs seem to invoke different speech recognition mechanisms: at the better SNR (4 dB), only vocabulary size played a role. This could be because speech intelligibility was relatively high in this condition for YNH (85.4% ± 10.6). Crucially, this condition showed the highest, albeit non-significant, correlation of working memory (RST) and speech recognition scores: listeners may have used the offline speech recognition strategy in this relatively easy condition. This observation is similar to the latent relation of working memory on speech recognition scores that was reported by Banks et al. (2015). At the lower SNR (6 dB), the combination of vocabulary size and lexical access time was important for speech recognition. This indicates that once perception becomes more difficult—as evidenced by lower speech recognition scores—efficient lexical access becomes more relevant.

For ONH, the picture is similar to YNH, but with slight differences: at the lower SNR (6), the combination of vocabulary size and lexical access time (M2), that is efficiency of lexical access, explains speech recognition, just as in the YNH group. At the better SNR (4 dB), none of our models including cognitive-linguistic factors, age, or hearing level could reliably model the subset data. We did, however, observe a trend for the combination of vocabulary size and lexical access time, suggesting a similar tendency for efficient lexical access as for YNH at the lower SNR (6 dB). Notably, ONH did not show any sign of the alternative ‘offline’ processing mechanism that we observed at 4 dB SNR in YNH. It thus appears that the speech recognition mechanisms used in noise change only slightly with age. Speech-in-noise recognition scores were nevertheless much lower for older compared to younger listeners. But neither working memory, as suggested by Van der Linden et al. (1994), Pichora-Fuller et al. (1995), and Benichov et al. (2012), nor pure-tone averages could account for this difference. It seems that efficiency of lexical access may be the best explanation for speech recognition in adverse conditions.

In summary, our results suggest that older adults with normal hearing apply mechanisms in speech recognition in noise that are very similar to those used by younger adults. It seems that lexical access time, possibly mediated by vocabulary size, is the most relevant correlate (or leading predictor). Although ONH were, on average, at least as good in their vocabulary size and working memory capacity as YNH, their lexical access times and speech recognition scores were worse. If there is, indeed an intricate interrelation between vocabulary size, working memory, and lexical access, as suggested by Banks et al. (2015), then a significant reduction in one of the three could arguably explain the poorer speech recognition scores. The fact that speech recognition in noise (at least for the lower SNR) in both YNH and ONH was modulated by the combination of vocabulary size and lexical access time (see the models M2 in **Table 6**) suggests that not accessing *speed* but accessing *efficiency* may be a relevant predictor. Following the argumentation of Ramscar et al. (2014), we assume that people with a larger mental lexicon require relatively longer search (accessing) times compared to people with smaller lexicons. If, however, people with large vocabularies are also fast in lexical access—which means their accessing efficiency is high—then this should result in a processing benefit and possibly better speech recognition results. If vocabulary size is somewhat comparable between groups but lexical decision time is considerably slowed in older adults, efficiency of lexical access may be affected. Our findings suggest that efficiency of lexical access declines with age, and this decline results in poorer speech recognition scores for ONH.

### Study Limitations

There are some noteworthy limitations to this study. Firstly, we observed a ceiling effect for the PPVT, especially in older adults, which could potentially have led to an underestimation of the role of vocabulary knowledge. We countered this effect by using a composite VOCABULARY factor that included both PPVT and WST scores, and by accounting for the LDT error rate for infrequent words. The latter were argued to reflect vocabulary size as well. Nevertheless, the influence of the composite VOCABULARY variable may possibly change as individual differences increase (but see Schmidtke, 2014; Kaandorp et al., 2015). Secondly, our measure for lexical access time was based on the simple RTs for existing words. These include the actual access, the search, but also decision times and general processing times, including motoric reaction of pressing a button. It is possible that the age-related differences partially pertain to general processing or motoric speed components. Future studies should therefore include a separate measure of processing speed to exclude motor or general processing speed and to allow a more “linguistic” interpretation. Thirdly, our two age groups were not completely equal in their hearing thresholds. Although preferable, a perfect match in pure-tone average was not feasible. We therefore cannot completely rule out any influence of hearing status (supra-threshold or otherwise), even though hearing level never turned up as significant predictor for speech in noise recognition. Since the focus of this study was mostly on aspects of speech recognition and the lexicon, we did not investigate any aspects of temporal or spectral coding of the signal and their relation to auditory processing. These latter aspects are, however, likely to decline with age as well, and have been shown to relate to reductions in speech-in-noise recognition scores (e.g., Füllgrabe et al., 2015; see also Rönnberg et al., 2013).

## Author Contributions

RC and AW conceptualized the research question, and the experimental design and conducted the measurements. RC analyzed the data and drafted the manuscript; RC and AW interpreted the results and wrote the manuscript. ER approved the experimental design, critically reviewed, and significantly contributed to the manuscript. BK provided framework for speech recognition tests and critically reviewed the manuscript. All authors approved the final version of the manuscript for publication. All authors agree to be accountable for all aspects of the work and in ensuring that questions related to the accuracy or integrity of any part of the work are appropriately investigated and resolved.

## Conflict of Interest Statement

The authors declare that the research was conducted in the absence of any commercial or financial relationships that could be construed as a potential conflict of interest.

The reviewer CS and handling Editor declared their shared affiliation, and the handling Editor states that the process nevertheless met the standards of a fair and objective review.

## Footnotes

1 http://wortschatz.uni-leipzig.de.

2 www.hoertech.de

## References

[B1] Akaike H. (1974). A new look at the statistical model identification. *IEEE Trans. Autom. Control.* 19 716–723. 10.1109/TAC.1974.1100705

[B2] Anderson S. Parbery-Clark A. Yi H.-G. Kraus N. (2011). A neural basis of speech-in-noise perception in older adults. *Ear Hear.* 32 750–757. 10.1097/AUD.0b013e31822229d3 21730859 PMC3189261

[B3] ANSI (1997). *American National Standard: Methods for Calculation of the Speech Intelligibility Index.* New York, NY: American National Stan- dards Institute.

[B4] ANSI (1999). ANSI/ASA S3.1-1999 (R2008): Maximum Permissible Ambient Noise Levels for Audiometric Test Rooms. Washington, DC: American National Standards Institute.

[B5] Baayen H. Milin P. (2010). Analyzing reaction times. *Int. J. Psychol. Res.* 3 12–28.

[B6] Baddeley A. D. Hitch G. (1974). “Working memory,” in *The Psychology of Learning and Motivation: Advances in Research and Theory* Vol. 8 ed. Bower G. H. (New York: Academic Press), 47–89.

[B7] Banks B. Gowen E. Munro K. J. Adank P. (2015). Cognitive predictors of perceptual adaptation to accented speech. *J. Acoust. Soc. Am.* 137 2015–2024. 10.1121/1.4916265 25920852

[B8] Bates D. Maechler M. Bolker B. Walker S. (2014). *lme4: Linear Mixed-Effects Models Using eigen 6 and S4. R Package Version 1.1- 7*, Available at: http://cran.r-project.org/package=lme4

[B9] Bell N. L. Lassiter K. S. Matthews T. D. Hutchinson M. B. (2001). Comparison of the peabody picture vocabulary test (3rd edition) and Wechsler adult intelligence scale (3rd edition) with university students. *J. Clin. Psychol.* 57 417–422. 10.1002/jclp.1024 11241372

[B10] Benard M. Mensink J. S. Başkent D. (2014). Individual differences in top-down restoration of interrupted speech: Links to linguistic and cognitive abilities. *J. Acoust. Soc. Am* 135 EL1–EL8 10.1121/1.4862879 25234920

[B11] Benichov J. Coy L. C. Tun P. Wingfield A. (2012). Word recognition within a linguistic context: effects of hearing acuity, verbal ability, and cognitive function. *Ear Hear.* 33 250–256. 10.1097/AUD.0b013e31822f680f 21918453 PMC3253325

[B12] Besser J. Festen J. M. Goverts S. T. Kramer S. E. Pichora-Fuller M. K. (2015). Speech-in-speech listening on the LiSN-S test by older adults with good audiograms depends on cognition and hearing acuity at high frequencies. *Ear Hear.* 36 24–41. 10.1097/AUD.0000000000000096 25207850

[B13] Besser J. Koelewijn T. Zekveld A. A. Kramer S. Festen J. (2013). How linguistic closure and verbal working memory relate to speech recognition in noise: a review. *Trends Amplif.* 17 75–93. 10.1177/1084713813495459 23945955 PMC4070613

[B14] Besser J. Zekveld A. A. Kramer S. Rönnberg J. Festen J. (2012). New measures of masked text recognition in relation to speech-in-noise perception and their association with age and cognitive abilities. *J. Speech Lang. Hear. Res.* 55 194–209. 10.1044/1092-4388(2011/11-0008) 22199191

[B15] Bharadwaj H. M. Masud S. Mehraei G. Verhulst S. Shinn-Cunningham B. (2015). Individual differences reveal correlates of hidden hearing deficits. *J. Neurosci.* 35 2161–2172. 10.1523/JNEUROSCI.3915-14.2015 25653371 PMC4402332

[B16] Bruce I. C. Legér A. C. Moore B. C. Lorenzi C. C. (2013). “Physiological prediction of masking release for normal-hearing and hearing-impaired listeners,” in *Proceeding of ICA*, (Canada: Montreal).

[B17] Buhlheller S. Häcker H. O. (2003). *Deutschsprachige Fassung des PPVT-III für Jugendliche und Erwachsene [German version of the PPVT-III for teenagers and adults].* Frankfurt: Swets.

[B18] Burke D. M. MacKay D. G. Worthley J. S. Wade E. (1991). On the tip of the tongue: what causes word finding failures in young and older adults? *J. Mem. Lang.* 30 542–579. 10.1016/0749-596X(91)90026-G

[B19] Carroll R. Meis M. Schulte M. Vormann M. Kießling J. Meister H. (2015). Development of a German Reading Span Test with dual task design for application in cognitive hearing research. *Int. J. Audiol.* 54 136–141. 10.3109/14992027.2014.952458 25195607

[B20] CHABA (1988). Speech understanding and aging. *J. Acoust. Soc. Am.* 83 859–895. 10.1121/1.395965 3281988

[B21] Cleland A. A. Gaskell M. G. Quinlan P. T. Tamminen J. (2006). Frequency effects in spoken and visual word recognition: evidence from dual-task methodologies. *J. Exp. Psychol. Hum. Percept. Perform.* 32 104–119. 10.1037/0096-1523.32.1.104 16478330

[B22] Cutler A. Clifton C. (2000). “Comprehending spoken language: a blueprint of the listener,” in *The Neurocognition of Language*, eds Brown C. M. Hagoort P. (Oxford: Oxford University Press), 123–166.

[B23] Desjardins J. L. Doherty K. A. (2013). Age-related changes in listening effort for various types of masker noises. *Ear Hear.* 34 261–272. 10.1097/AUD.0b013e31826d0ba4 23095723

[B24] Dirks D. D. Takayanagi S. Moshfegh A. (2001). Effects of lexical factors on word recognition among normal-hearing and hearing-impaired listeners. *J. Am. Acad. Audiol.* 12 233–244. 11392435

[B25] Dubno J. R. Dirks D. D. Morgan D. E. (1984). Effects of age and mild hearing loss on speech recognition in noise. *J. Acoust. Soc. Am.* 76 87–96. 10.1121/1.391011 6747116

[B26] Dubno J. R. Horwitz A. R. Ahlstrom J. B. (2002). Benefit of modulated maskers for speech recognition by younger and older adults with normal hearing. *J. Acoust. Soc. Am.* 111 2897–2907. 10.1121/1.1480421 12083223

[B27] Füllgrabe C. (2015). Beyond audibility – the role of supra-threshold auditory processing and cognition in presbycusis. *Hear. Rev.* 22:18.

[B28] Füllgrabe C. Moore B. C. (2014). Effects of age and hearing loss on stream segregation based on interaural time differences. *J. Acoust. Soc. Am.* 136 EL185–EL191 10.1121/1.4890201 25096145

[B29] Füllgrabe C. Moore B. C. J. Stone M. A. (2015). Age-group differences in speech identification despite matched audiometrically normal hearing: contributions from auditory temporal processing and cognition. *Front. Aging Neurosci.* 6:347 10.3389/fnagi.2014.00347 PMC4292733 25628563

[B30] George E. L. J. Festen J. M. Houtgast T. (2006). Factors affecting masking release for speech in modulated noise for normal-hearing and hearing-impaired listeners. *J. Acoust. Soc. Am.* 120 2295–2311. 10.1121/1.2266530 17069325

[B31] Hartshorne J. Germine L. T. (2015). When does cognitive function peak? The asynchronous rise and fall of different cognitive abilities across the life span. *Psychol. Sci.* 26 433–443. 10.1177/0956797614567339 25770099 PMC4441622

[B32] Heinrich A. Henshaw H. Ferguson M. A. (2015). The relationship of speech intelligibility with hearing sensitivity, cognition, and perceived hearing difficulties varies for different speech perception tests. *Front. Psychol. Aud. Cogn. Neurosci.* 6:782 10.3389/fpsyg.2015.00782 PMC4468362 26136699

[B33] Huettig F. Janse E. (2016). Individual differences in working memory and processing speed predict anticipatory spoken language processing in the visual world. *Lang. Cogn. Neurosci.* 31 80–93. 10.1080/23273798.2015.1047459

[B34] Humes L. E. Dubno J. R. Gordon-Salant S. Lister J. J. Cacace A. T. Cruickshanks K. J. (2012). Central presbycusis: a review and evaluation of the evidence. *J. Am. Acad. Audiol.* 23 635–666. 10.3766/jaaa.23.8.5 22967738 PMC5898229

[B35] ISO (2004). *ISO 389 - 8:2004: Acoustics - Reference Zero for the Calibration of Audiometric Equipment - Part 8: Reference Equivalent Threshold Sound Pressure Levels for Pure Tones and Circumaural Earphones.* Geneva: International Organization for Standardization.

[B36] Janse E. (2009). Processing of fast speech by elderly listeners. *J. Acoust. Soc. Am.* 125 2361–2373. 10.1121/1.3082117 19354410

[B37] Kaandorp M. W. de Groot A. M. B. Festen J. M. Smits C. Goverts S. T. (2015). The influence of lexical-access ability and vocabulary knowledge on measures of speech recognition in noise. *Int. J. Audiol.* 55 157–167. 10.3109/14992027.2015.1104735 26609557

[B38] Kavé G. Halamish V. (2015). Doubly blessed: older adults know more vocabulary and know better what they know. *Psychol. Aging* 30 68–73. 10.1037/a0038669 25602490

[B39] Kavé G. Knafo A. Gilboa A. (2010). The rise and fall of word retrieval across the lifespan. *Psychol. Aging* 25 719–724. 10.1037/a0018927 20853975

[B40] Kavé G. Yafé R. (2014). Performance of younger and older adults on tests of word knowledge and word retrieval: independence or interdependence of skills? *Am. J. Speech Lang. Pathol.* 23 36–45. 10.1044/1058-0360(2013/12-0136) 23831710

[B41] Kemper S. Sumner A. (2001). The structure of verbal abilities in young and older adults. *Psychol. Aging* 16 312–322. 10.1037/0882-7974.16.2.312 11405318

[B42] Keuleers E. Stevens M. Mandera P. Brysbaert M. (2015). Word knowledge in the crowd: measuring vocabulary size and word prevalence in a massive online experiment. *Q. J. Exp. Psychol.* 68 1665–1692. 10.1080/17470218.2015.1022560 25715025

[B43] Kidd G. R. Humes L. E. (2015). Keeping track of who said what: performance on a modified auditory n-back task with young and older adults. *Front. Psychol.* 6:987 10.3389/fpsyg.2015.00987 PMC4510348 26257666

[B44] Kollmeier B. Wesselkamp T. (1997). Development and evaluation of a German sentence testfor objective and subjective speech intelligibility assessment. *J. Acoust. Soc. Am.* 102 2412–2421. 10.1121/1.419624 9348699

[B45] Legér A. C. Moore B. C. J. Lorenzi C. (2012). Temporal and spectral masking release in low- and mid-frequency regions for normal-hearing and hearing-impaired listeners. *J. Acoust. Soc. Am.* 131 1502–1514. 10.1121/1.3665993 22352520

[B46] MacKay D. G. (1987). *The Organization of Perception and Action: A Theory for Language and Other Cognitive Skills.* New York, NY: Springer.

[B47] MacKay D. G. Burke D. M. (1990). “Cognition and aging: a theory of new learning and the use of new connections,” in *Aging and Cognition: Knowledge Organization and Utilization*, ed. Hess T. M. (Amsterdam: North Holland), 213–262.

[B48] Mackersie C. L. MacPhee I. X. Heldt E. W. (2015). Effects of hearing loss on heart rate variability and skin conductance measured during sentence recognition in noise. *Ear Hear.* 36 145–154. 10.1097/AUD.0000000000000091 25170782 PMC4272605

[B78] Marslen-Wilson W. D. (ed.). (1989). “Access and integration: projecting sound onto meaning,” in *Lexical Representation and Process* (Cambridge, MA: MIT Press).

[B49] McAuliffe M. J. Gibson E. M. Kerr S. E. Anderson T. LaShell P. J. (2013). Vocabulary influences older and younger listeners’ processing of dysarthric speech. *J. Acoust. Soc. Am.* 134 1358–1368. 10.1121/1.4812764 23927132

[B50] Mishra S. Lunner T. Stenfelt S. Rönnberg J. Rudner M. (2013). Visual information can hinder working memory processing of speech. *J. Speech Lang. Hear. Res.* 56 1120–1132. 10.1044/1092-4388(2012/12-0033) 23785180

[B51] Mishra S. Stenfelt S. Lunner T. Rönnberg J. Rudner M. (2014). Cognitive spare capacity in older adults with hearing loss. *Front. Aging Neurosci.* 6:96 10.3389/fnagi.2014.00096 PMC4033040 24904409

[B52] Neger T. M. Rietveld T. Janse E. (2014). Relationship between perceptual learning in speech and statistical learning in younger and older adults. *Front. Hum. Neurosci.* 8:628 10.3389/fnhum.2014.00628 PMC4150448 25225475

[B53] Nilsson M. Solí S. D. Sullivan J. A. (1994). Development of the hearing in noise test for the measurement of speech reception thresholds in quiet and in noise. *J. Acoust. Soc. Am.* 95 1085–1099. 10.1121/1.408469 8132902

[B54] Pichora-Fuller M. K. (2003). Cognitive aging and auditory information processing. *Int. J. Audiol.* 42 S26–S32. 10.3109/14992020309074641 12918626

[B55] Pichora-Fuller M. K. Souza P. E. (2003). Effects of aging on auditory processing of speech. *Int. J. Audiol.* 42 S11–S16. 10.3109/14992020309074641 12918623

[B56] Pichora-Fuller M. K. Scheider B. A. Daneman M. (1995). How young and old adults listen to and remember speech in noise. *J. Acoust. Soc. Am.* 97 593–608. 10.1121/1.412282 7860836

[B57] Picou E. M. Ricketts T. A. Hornsby B. W. (2013). How hearing aids, background noise, and visual cues influence objective listening effort. *Ear. Hear.* 34 e52–e64. 10.1097/AUD.0b013e31827f0431 23416751

[B58] Plomp R. Mimpen A. M. (1979). Improving the reliability of testing the speech reception threshold for sentences. *Audiology* 18 43–52. 10.3109/00206097909072618 760724

[B59] Ramscar M. Hendrix P. Shaoul C. Milin P. Baayen H. (2014). The myth of cognitive decline: non-linear dynamics of lifelong learning. *Top. Cogn. Sci.* 6 5–42. 10.1111/tops.12078 24421073

[B60] Rönnberg J. Lunner T. Zekveld A. A. Sörqvist P. Danielson H. Lyxell B. (2013). The ease of language understanding (ELU) model: theoretical, empirical, and clinical advances. *Front. Sys. Neurosci.* 7:31 10.3389/fnsys.2013.00031 PMC3710434 23874273

[B61] Rudner M. Lunner T. Behrens T. Thorén E. S. Rönnberg J. (2012). Working memory capacity may influence perceived effort during aided speech recognition in noise. *J. Am. Acad. Audiol.* 23 577–589. 10.3766/jaaa.23.7.7 22967733

[B62] Rudner M. Signoret C. (2016). The role of working memory and executive function in communication under adverse conditions. *Front. Psychol.* 7:148 10.3389/fpsyg.2016.00148 PMC4749708 26903938

[B63] Salthouse T. A. (2004). What and when of cognitive aging. *Curr. Dir. Psychol. Sci.* 13 140–144. 10.1111/j.0963-7214.2004.00293.x

[B64] Salthouse T. A. (2010). *Major Issues in Cognitive Aging.* New York, NY: Oxford University Press.

[B65] Schmidt K.-H. Metzler P. (1992). *Wortschatztest.* Weinheim: Beltz.

[B66] Schmidtke J. (2014). Second language experience modulates word retrieval effort in bilinguals: evidence from pupillometry. *Front. Psychol.* 5:137 10.3389/fpsyg.2014.00137 PMC3930865 24600428

[B67] Schoof T. Rosen S. (2014). The role of auditory and cognitive factors in understanding speech in noise by normal-hearing older listeners. *Front. Aging Neurosci.* 6:307 10.3389/fnagi.2014.00307 PMC4228854 25429266

[B68] Steeneken H. J. M. Houtgast T. (1980). A physical method for measuring speech-transmission quality. *J. Acoust. Soc. Am.* 67 318–326. 10.1121/1.384464 7354199

[B69] Stone A. M. Moore B. C. J. (2014). Amplitude-modulation detection by recreational-noise-exposed humans with near-normal hearing thresholds and its medium-term progression. *Hear. Res.* 317 50–62. 10.1016/j.heares.2014.09.005 25260433 PMC4228076

[B70] Uslar V. N. Ruigendijk E. Hamann C. Brand T. Kollmeier B. (2011). How does linguistic. *Int. J. Audiol.* 50 621–631. 10.(3109)/14992027.2011.582166 21714708

[B71] Van der Linden M. Brédart S. Beerten A. (1994). Age-related differences in updating working memory. *Br. J. Psychol.* 85 145–152. 8167975 10.1111/j.2044-8295.1994.tb02514.x

[B72] Vitevitch M. S. Luce P. (1998). When words compete: levels of processing in perception of spoken words. *Psychol. Sci.* 9 325–329. 10.1111/1467-9280.00064

[B73] Wayne R. V. Johnsrude I. (2015). A review of causal mechanisms underlying the link between age-related hearing loss and cognitive decline. *Ageing Res. Rev.* 23 154–166. 10.1016/j.arr.2015.06.002 26123097

[B74] Weber A. Scharenborg O. (2012). Models of spoken-word recognition. *WIREs Cogn. Sci.* 3 387–401. 10.1002/wcs.1178 26301470

[B75] Wingfield A. Amichetti N. Lash A. (2015). Cognitive aging and hearing acuity: modeling spoken language comprehension. *Front. Psychol.* 6:684 10.3389/fpsyg.2015.00684 PMC4462993 26124724

[B76] World Health Organization [WHO] (2016). *Grades of Hearing Impairment.* Available at: http://www.who.int/pbd/deafness/hearing_impairment_grades/en/

[B77] Zekveld A. A. Kramer S. E. Festen J. M. (2011). Cognitive load during speech perception in noise: the influence of age, hearing loss, and cognition on the pupil response. *Ear Hear.* 32 498–510. 10.1097/AUD.0b013e31820512bb 21233711

